# Impropriety of Frequent Operative Interference in Midwifery

**Published:** 1850-05

**Authors:** R. Collins

**Affiliations:** Dublin


*Impropriety of Frequent Operative Interference in Midwifery.* By Dr. R. Collins, Dublin.

[Dr. Collins gives an account of 3847 labors attended by the late Dr. Joseph Clarke, of Dublin, in which he used instruments remarkably seldom. Dr. Collins says:]

It may be observed he only used the forceps once, and that without completing the delivery. If we seriously reflect upon the happy results to the mothers, from the practice pursued by this distinguished physician, as regards the use of instruments, and then carefully examine the succeeding section upon children still born, and find here equally happy results (as of the 3816 single births, there was only forty-two children still-born, of those that had arrived at the full period of gestation, or in the singularly small proportion of one in 91), we cannot fail to discover a number of astounding truths, sufficient to warn our artificial advocates, and make them pause until they can supply their professional brethren with a series of facts equally satisfactory.

How seldom should most practitioners be found to use instruments, if the successful course pursued by Doctor Clarke were universally aimed at. Is it not worthy of our best consideration, with the invaluable statement before us, that, in an extended practice in the upper ranks of life perhaps unexampled, there is not *one single instance of death* resulting from laborious or protracted labor! This is a practical fact, which ought to be carefully recollected, and seriously weighed, by most of our continental brethren, who use instruments in every fifth, tenth, fifteenth, twentieth, or thirtieth labor under their care, with the object of expediting delivery; as also by some of our own countrymen, whose unsound doctrines, inculcating mischievous interference to promote hasty delivery, the unquestionable truths here recorded clearly demonstrate to be most unjustifiable and most uncalled for. Should not this inexpressibly important record forever silence those who venture to publish crude and fanciful opinions, unsupported by any data from their own experience, affording similarly happy results. It affords me infinite satisfaction to supply this truthful registry of facts for the universal and serious consideration of the profession. These truths speak in language the most convincing, and must, when studied, leave an indelible impression.—*Med. Gaz., July* 20, 1849, *p.* 122.

